# Complete Genome Sequence of *Campylobacter iguaniorum* Strain RM11343, Isolated from an Alpaca

**DOI:** 10.1128/genomeA.00646-16

**Published:** 2016-06-30

**Authors:** William G. Miller, Emma Yee, Stephen Huynh, Mary H. Chapman, Craig T. Parker

**Affiliations:** Produce Safety and Microbiology Research Unit, Agricultural Research Service, U.S. Department of Agriculture, Albany, California, USA

## Abstract

*Campylobacter iguaniorum* is a member of the *C. fetus* group of campylobacters and is one of two *Campylobacter* taxa isolated from reptiles. This study describes the whole-genome sequence of the *C. iguaniorum* strain RM11343, which was isolated from a California alpaca fecal sample.

## GENOME ANNOUNCEMENT

*Campylobacter* spp. are isolated typically from a wide variety of warm-blooded animals and birds. However, two *Campylobacter* taxa, *C. fetus* subsp. *testudinum* (1) and *C. iguaniorum* (2), have been isolated from reptiles. These organisms have been isolated from lizards, snakes, and chelonians (1–6) and occasionally cause disease in humans (1, 7, 8). In 2010, a *C. iguaniorum* strain (RM11343) was isolated in California from an alpaca fecal sample. This study presents the genome sequence of *C. iguaniorum* strain RM11343, the first strain of this species isolated outside of reptiles.

The Roche GS-FLX and Illumina MiSeq platforms were used to complete the RM11343 genome. A total of 159,788 shotgun and paired-end Roche 454 reads (52× coverage) were assembled, using the Roche Newbler assembler version 2.6, into a single scaffold of 13 contigs. All 454 base calls were validated using 2,193,386 Illumina MiSeq reads, adding 416× coverage. Contigs that spanned the scaffold gaps and the MiSeq reads were used to close the scaffold into a single contig. An optical restriction map (OpGen, Gaithersburg, MD, USA) with the restriction enzyme AflII was used to validate the assembly. Illumina MiSeq reads were also used to characterize hypervariable GC tracts, as described (9).

*C. iguaniorum* strain RM11343 has a circular genome of 1,544 kb with a GC content of 35.8%. Protein-, rRNA-, and tRNA-encoding genes were identified as described (9), but using a BLASTp identity of 40% to define a positive match. The genome encodes 1,487 putative protein-coding genes, 86 pseudogenes, 44 tRNAs, and 3 rRNA operons. The RM11343 genome also contains 14 hypervariable homopolymeric GC tracts (≥8 bp). No plasmids were identified. The genomes of the *C. iguaniorum* reptile strains 1485E and 2463D (GenBank accession numbers CP009043 and CP010995, respectively) were used in all comparative genomic analyses.

The average nucleotide identity of strain RM11343 when compared to the *C. iguaniorum* strains 1485E and 2463D is 98%, which is consistent with its *C. iguaniorum* identification. Furthermore, the GC content (35.8%) is identical to the average GC content of the reptile-associated strains. The three *C. iguaniorum* genomes are also highly syntenic with very similar gene content: 1,344/1,487 (90%) of the proteins encoded by RM11343 are also present in the proteomes of both 1485E and 2463D. Many of the remaining 10% are encoded by genes or regions typically variable in *Campylobacter* spp. (e.g., R/M systems and genetic islands).

Despite the similarities between the *C. iguaniorum* genomes, the RM11343 genome contains two noteworthy genomic islands (GIs) not present within strains 1485E or 2463D. The first is a zonula occludens toxin GI, previously identified within *Campylobacter* spp. (10–12) and other pathogens (13). The second GI contains a *kps*/*neu* capsular locus similar to that identified in *Escherichia coli* K1 (14). This locus, bounded by *kpsMTED* and *kpsCS*, contains a putative *neuBCAES* gene cluster that would encode the α-2,8-polysialyltransferase necessary for biosynthesis of polysialic acid. The presence of these two GIs warrants further investigation into the pathogenicity of this strain.

### Nucleotide sequence accession number.

The complete genome sequence of *C. iguaniorum* strain RM11343 has been deposited in GenBank under the accession number CP015577.
